# Food recalls associated with foodborne disease outbreaks, United States, 2006–2016

**DOI:** 10.1017/S0950268821001722

**Published:** 2021-07-19

**Authors:** Qihua Qiu, Daniel Dewey-Mattia, Sanjana Subramhanya, Zhaohui Cui, Patricia M. Griffin, Susan Lance, William Lanier, Matthew E. Wise, Samuel J. Crowe

**Affiliations:** 1 Augusta University, Augusta, GA30912, USA; 2 Centers for Augusta University Disease Control and Prevention, Augusta, GA30912,USA; 3 Food and Drug Administration, Silver Spring, MD, USA; 4 USDA Food Safety and Inspection Service, Washington, DC, USA

**Keywords:** Food safety, food-borne infections, outbreaks, recalls

## Abstract

About 800 foodborne disease outbreaks are reported in the United States annually. Few are associated with food recalls. We compared 226 outbreaks associated with food recalls with those not associated with recalls during 2006–2016. Recall-associated outbreaks had, on average, more illnesses per outbreak and higher proportions of hospitalisations and deaths than non-recall-associated outbreaks. The top confirmed aetiology for recall-associated outbreaks was *Salmonella*. Pasteurised and unpasteurised dairy products, beef and molluscs were the most frequently implicated foods. The most common pathogen−food pairs for outbreaks with recalls were *Escherichia coli*-beef and norovirus-molluscs; the top pairs for non-recall-associated outbreaks were scombrotoxin-fish and ciguatoxin-fish. For outbreaks with recalls, 48% of the recalls occurred after the outbreak, 27% during the outbreak, 3% before the outbreak, and 22% were inconclusive or had unknown recall timing. Fifty per cent of recall-associated outbreaks were multistate, compared with 2% of non-recall-associated outbreaks. The differences between recall-associated outbreaks and non-recall-associated outbreaks help define the types of outbreaks and food vehicles that are likely to have a recall. Improved outbreak vehicle identification and traceability of rarely recalled foods could lead to more recalls of these products, resulting in fewer illnesses and deaths.

## Introduction

Foodborne illness is an important cause of morbidity and mortality in the United States [1]. Although only a small proportion of foodborne illnesses are associated with recognised outbreaks, outbreak investigations provide valuable epidemiological and risk mitigation information about the foods and pathogens that cause disease [2]. When outbreaks occur, investigators aim to identify the contaminated food as quickly as possible to prevent additional illnesses. One tool sometimes used when a contaminated food is identified is a food recall, which is an action taken by a food facility to remove a food product from commerce when evidence indicates a link between illness and a particular food [3]. A recall is accompanied by a published notification from a regulatory agency that stipulates that a food might be unsafe and that it should be returned or discarded. It also may entail a similar notice from a company. Food recalls are categorised into three classes depending on the risk to the public associated with the contaminated food [3, 4]. Two federal regulatory agencies, the U.S. Food and Drug Administration (FDA) and the U.S. Department of Agriculture's Food Safety and Inspection Service (FSIS), are involved in this process and can request that a company recall a food product.

We summarised foodborne disease outbreaks reported to the U.S. Centers for Disease Control and Prevention (CDC) during 2006–2016 associated with food recalls and compared them with outbreaks that did not result in a recall in order to better understand the differences between outbreaks with and without recalls and to identify characteristics of outbreaks that might be related to the likelihood of a food recall.

## Methods

We used 2006–2016 data from the Foodborne Disease Outbreak Surveillance System (FDOSS), a CDC surveillance system that collects reports of foodborne disease outbreaks occurring in the United States. A foodborne disease outbreak was defined as two or more cases of similar illness due to ingestion of a common food. We classified each outbreak that was linked to a recall as a recall-associated outbreak. If two outbreaks were linked to the same recall, we analysed them as two outbreaks and the food vehicle was counted twice. If the exposure occurred in one state, the outbreak was classified as a single state outbreak; if the exposure occurred in more than one state, the outbreak was classified as a multistate outbreak. We excluded outbreaks caused by a sick food handler because they are usually due to contamination at the point of service, such as at a restaurant, and, thus, are rarely associated with a product recall.

Aetiologies were classified as bacterial, viral, parasitic, chemical/toxin or multiple, and were further classified as confirmed or suspected based on established criteria set by CDC [5]. Outbreaks were considered to have multiple confirmed aetiologies when two or more aetiology types were reported and confirmed. They were considered to have multiple suspected aetiologies if multiple aetiologies were reported but no more than one was confirmed.

Food vehicles were categorised using a hierarchical scheme that categorises foods implicated in outbreaks with increasing specificity at each level [6]. We stratified foods into 24 categories, including fish, beef, dairy and fruits.

For recalls linked to outbreaks, we analysed the timing of food recalls relative to illness onset dates. When a recall date was missing from the dataset, we searched outbreak web postings and FDA or FSIS news releases to identify it. Recalls were grouped by date into five categories: recalls that occurred (1) before the date of first reported illness onset (before the outbreak); (2) after the date of first reported illness onset but before the last illness onset (during the outbreak); (3) after the last illness onset (after the outbreak); (4) after the date of first illness onset but uncertain if before the last illness onset (inconclusive); and (5) unknown.

## Results

**Fig. 1.**
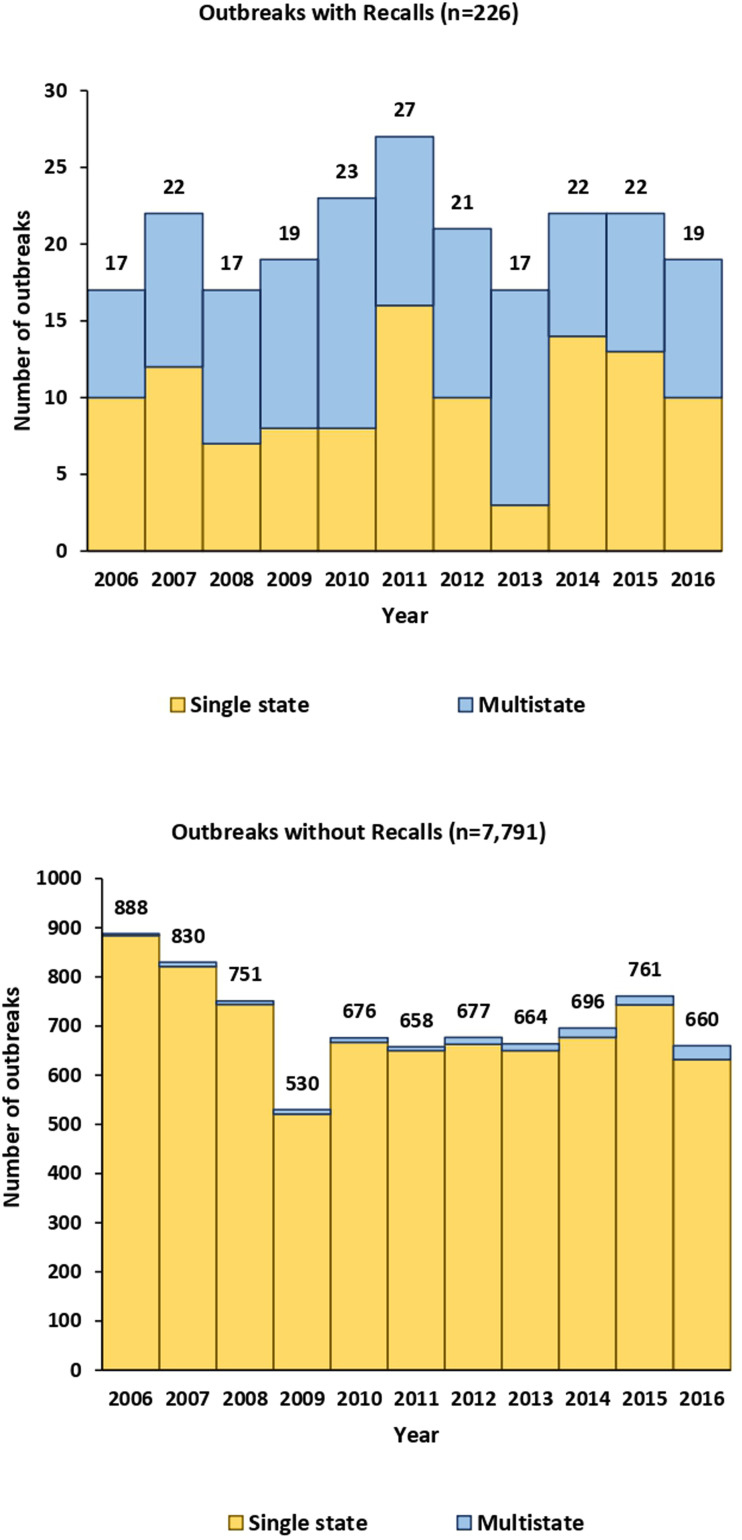
Outbreaks by recall status, outbreak type and year of illness onset, foodborne disease outbreak surveillance system, United States, 2006–2016.

During 2006–2016, 8017 foodborne disease outbreaks that did not involve a sick food handler were reported, comprising 140 917 illnesses, 8756 hospitalisations and 204 deaths. Of these outbreaks, 226 (3%) were associated with 219 food recalls; in seven instances, two outbreaks were associated with the same food recall. A total of 16 106 illnesses, 2875 hospitalisations and 111 deaths were linked to recall-associated outbreaks. On average, recall-associated outbreaks had 71 illnesses per outbreak, a hospitalisation rate of 28.3% and a death rate of 2.6%. In comparison, non-recall-associated outbreaks had 16 illnesses per outbreak, a hospitalisation rate of 8.1% and a death rate of 0.2% (Table 1). An average of 20 recall-associated outbreaks were reported each year (in comparison to an average of 708 non-recall outbreaks per year), ranging from 17 in 2006, 2008 and 2013, to 27 in 2011 (Fig. 1). The annual number of illnesses associated with recall-associated outbreaks ranged from a high of 3084 cases in 2006 to a low of 246 in 2014.

**Table 1.** Comparison of illnesses, hospitalisations and deaths by outbreak type and recall status, foodborne disease outbreak surveillance system, United States, 2006–2016

	Single state		Multistate		Total	
	with recalls	Without recalls	with recalls	Without recalls	with recalls	Without recalls
Outbreaks	111	7652	115	139	226	7791
Illnesses	3891	119 699	12 215	5112	16 106	124 811
Hospitalisations	319	5067	2556	814	2875	5881
Deaths	12	84	99	9	111	93
Illnesses per outbreak						
Mean	35	16	106	37	71	16
Median	9	7	25	20	17	7
Range	2**–**1644	2**–**802	2**–**1939	2**–**275	2**–**1939	2**–**802
Hospitalisation rate						
Mean (%)	20.3	7.8	36.8	23.1	28.3	8.1
Median (%)	4.7	0	27.3	18.8	18.2	0
Range (%)	0**–**100	0**–**100	0**–**100	0**–**100	0**–**100	0**–**100
Death rate						
Mean (%)	1.6	0.2	3.7	0.9	2.6	0.2
Median (%)	0	0	0	0	0	0
Range (%)	0**–**50	0**–**100	0**–**50	0**–**50	0**–**50	0**–**100

Of recall-associated outbreaks, 97% (219/226) had aetiologies reported (Table 2). Among confirmed, single-aetiology, recall-associated outbreaks, 83% (170/205) were caused by bacteria. The top three causes were *Salmonella* (67 outbreaks; including nine outbreaks of *S.* Enteritidis, eight of *S*. Typhimurium and seven of *S*. Newport), *E. coli* (56 outbreaks; including 47 of Shiga toxin-producing *Escherichia coli* (STEC) O157, four of STEC O26 and two of STEC O121) and *Listeria monocytogenes* (25 outbreaks). In comparison, the top three pathogens for non-recall outbreaks with a confirmed aetiology were *Salmonella* (1176 outbreaks; including 304 of *S*. Enteritidis, 153 of *S*. Typhimurium and 105 of *S*. Newport), norovirus (1113 outbreaks) and *E*. *coli* (244 outbreaks; including 190 of STEC O157, 12 of STEC O26 and eight of STEC O121). The pathogens from confirmed-aetiology outbreaks with the highest proportion of recalls were *Listeria monocytogenes* (25/48, 52%), *E. coli* (56/300, 19%), *Vibrio* (8/54, 15%) and hepatitis A virus (3/20, 15%).

**Table 2.** Outbreaks by aetiology and recall status, foodborne disease outbreak surveillance system, United States, 2006–2016^a^

Aetiology	Outbreaks								
	with recalls				Without recalls				Total^b^(*n*)
	*n*			%^c^	*n*			%^c^	
	CE^d^	SE^d^	Total		CE^d^	SE^d^	Total		
Bacterial									
*Salmonella*	67	0	67	33	1176	68	1244	33	1311
*E. coli*	56	0	56	27	244	22	266	7	322
*Listeria*	25	1	26	12	23	0	23	1	49
*Campylobacter*	8	0	8	4	220	65	285	1	293
*Vibrio*	8	2	10	4	46	23	69	6	79
*Clostridium botulinum*	3	0	3	1	28	4	32	1	35
*Clostridium perfringens*	1	0	1	<1	188	150	338	5	339
*Bacillus*	2	0	2	1	35	81	116	1	118
*Shigella*	0	0	0	0	45	9	54	1	54
*Staphylococcus*	0	0	0	0	40	61	101	1	101
Other bacterium	0	1	1	0	12	62	74	<1	75
Subtotal	170	4	174	83	2057	545	2602	58	2776
Viral									
Norovirus	15	7	22	7	1113	875	1988	39	2010
Hepatitis A	3	0	3	1	17	0	17	<1	20
Rotavirus	0	0	0	0	2	0	2	<1	2
Sapovirus	0	0	0	0	4	1	5	<1	5
Other virus	0	0	0	0	2	3	5	<1	5
Subtotal	18	7	25	9	1138	879	2017	32	2042
Parasitic									
*Cryptosporidium*	0	0	0	0	18	3	21	1	21
*Cyclospora*	0	0	0	0	14	0	14	<1	14
*Giardia*	0	0	0	0	6	2	8	<1	8
*Trichinella*	0	0	0	0	12	1	13	<1	13
Other parasite	0	0	0	0	1	0	1	<1	1
Subtotal	0	0	0	0	51	6	57	1	57
Chemical or toxin									
Scombrotoxin	8	1	9	4	156	11	167	4	176
Ciguatoxin	2	0	2	1	123	16	139	3	141
Other chemical/toxin	7	2	9	3	58	56	114	2	123
Subtotal	17	3	20	8	337	83	420	9	440
***Single aetiology***	205	14	219	100	3583	1513	5096	98	5315
***Multiple aetiologies***	0	0	0	0	36	100	136	2	136
***Unknown aetiology***	0	7	7	0	0	2559	2559	0	2566
***Total***^e^	205	21	226	100	3619	4172	7791	100	8017

a Outbreaks caused by a sick food handler are excluded.

b Total number includes all of the confirmed and suspected aetiology outbreaks for each aetiology.

c % of all confirmed single-aetiology outbreaks.

d CE, confirmed aetiology; SE, suspected aetiology.

e Total number includes all the outbreaks caused by a single aetiology, multiple aetiologies and unknown aetiology. All food categories (a single food category and multiple food categories) are included.

Among recall-associated outbreaks, 88% (199/226) had vehicles that could be classified into a single food category, 7% (15/226) had food vehicles that could be classified into multiple food categories, and 5% (12/226) were caused by an unknown (i.e. unreported) food (Table 3). Pasteurised and unpasteurised dairy products (33, 17%), beef (32, 16%) and molluscs (30, 15%) were the most frequently implicated foods in recall-associated outbreaks. Among the 30 recall-associated outbreaks due to molluscs, 28 were linked to oysters. As a comparison, fish (346, 20%), dairy products (174, 10%) and chicken (172, 10%) were the most frequently implicated foods in non-recall outbreaks. The food categories with the highest proportion of outbreaks with recalls were nuts and seeds (12/18 outbreaks, 67%), herbs (5/9, 56%), sprouts (16/31, 52%) and molluscs (30/154, 19%).

**Table 3.** Outbreaks by food category and recall status, foodborne disease outbreak surveillance system, United States, 2006–2016^a^

Food category	Outbreaks					
	with recalls		Without recalls		Total (*n*)	Recall/Total (%)
	*n*	%	*n*	%		
Aquatic animals						
Fish	13	7	346	20	359	4
Molluscs	30	15	124	7	154	19
Crustaceans	0	0	28	2	28	0
Other aquatic animals	0	0	7	0	7	0
Subtotal	43	22	505	30	548	8
Land animals						
Beef	32	16	160	9	192	17
Pork	4	2	126	7	130	3
Other meat	0	0	8	0	8	0
Chicken	6	3	172	10	178	3
Turkey	2	1	67	4	69	3
Other poultry	0	0	8	0	8	0
Game	1	1	15	1	16	6
Eggs	5	3	50	3	55	9
Dairy	33	17	174	10	207	16
Subtotal	83	42	780	46	863	10
Plants						
Vegetable row crops	8	4	74	4	82	10
Seeded vegetables	6	3	53	3	59	10
Root/Underground	1	1	24	1	25	4
Sprouts	16	8	15	1	31	52
Herbs	5	3	4	0	9	56
Fungi	0	0	25	1	25	0
Grains/Beans	3	2	81	5	84	4
Nuts/Seeds	12	6	6	0	18	67
Oils/Sugars	0	0	6	0	6	0
Fruits	17	9	74	4	91	19
Subtotal	68	34	362	21	430	16
***Other***	5^b^	3	51	3	56	9
***Total single food category***	199	88	1698	22	1897	10
***Multiple food categories***	15	7	1337	17	1352	1
***Unknown food category***	12	5	4756	61	4768	<1
***Total***^c^	226	100	7791	100	8017	3

a Outbreaks caused by a sick food handler are excluded.

b The sources of the five recall-associated outbreaks were sindoor, a flavoured drink, dietary supplements (2 outbreaks) and seaweed.

c Total includes all outbreaks associated with a single food category, multiple food categories and unknown food category of any aetiology type (single, multiple, or unknown).

There were considerable differences between some of the top pathogen-food pairs for outbreaks with recalls compared with those without recalls. The most common pairs for outbreaks with recalls were *E. coli* and beef and norovirus and molluscs, but the top pairs for non-recall-associated outbreaks were scombrotoxin and fish and ciguatoxin and fish (Table 4). Among the 62 confirmed *Salmonella* outbreaks linked to a single food category associated with a recall, sprouts (11 outbreaks, 18%), nuts and seeds (11, 18%), fruits (6, 10%) and chicken (6, 10%) were the top food vehicles, but the top three food vehicles for the 340 confirmed *Salmonella* single-food category outbreaks that did not have a recall were chicken (60, 18%), pork (50, 15%) and eggs (43, 13%) (Table 5). In addition to *Salmonella*, some pathogen-food pairs, such as *Campylobacter* and dairy and *Clostridium perfringens* and beef, were frequent combinations in outbreaks, but rarely resulted in recalls. Confirmed norovirus single-food category outbreaks that resulted in a recall (*n* = 15) were all due to molluscs (e.g. oysters), but there were a variety of food vehicles for confirmed norovirus single-food category outbreaks without recalls (*n* = 74), including vegetable row crops (e.g. lettuce, celery) (17, 23%), molluscs (13, 18%) and fruits (e.g. cantaloupe, berries) (11, 15%) (Table 5).

**Table 4.** Top 10 confirmed single aetiology and food combinations by recall status, foodborne disease outbreak surveillance system, United States, 2006–2016

With recalls		Without recalls	
Pathogen food	No. of outbreaks	Pathogen-food	No. of outbreaks
*E. coli* – Beef	27	Scombrotoxin – Fish	143
Norovirus – Molluscs	15	Ciguatoxin – Fish	114
*Listeria* – Dairy	13	*Campylobacter* – Dairy	81
*Salmonella* – Nuts-Seeds	11	*Salmonella* – Chicken	60
*Salmonella* – Sprouts	11	*Vibrio* – Molluscs	40
*E. coli* – Dairy	9	*Salmonella* – Pork	50
Scombrotoxin – Fish	8	*Salmonella* – Eggs	43
*Vibrio* – Molluscs	8	*E. coli* – Beef	39
*Salmonella* – Chicken	6	*Salmonella* – Seeded vegetables	31
*Campylobacter* – Dairy	6	*Clostridium perfringens* – Beef	28
***Subtotal***	114		629
***Other identified combinations***	72		522
***Total identified combinations***	186		1151

*Note*: This table includes only outbreaks caused by a confirmed single aetiology and a single food category. Outbreaks caused by a sick food handler are excluded.

**Table 5.** Top Confirmed Single Aetiologies Implicated by Single Food Categories and Recall Status, Foodborne Disease Outbreak Surveillance System, United States, 2006–2016^a^

With Recalls			Without Recalls		
	*n*	(%)		*n*	(%)
*E. coli*			*Salmonella*		
Beef	27	(52)	Chicken	60	(18)
Dairy	9	(17)	Pork	50	(15)
Vegetable row crops	6	(12)	Eggs	43	(13)
Fruits	4	(8)	Seeded vegetables	31	(9)
Sprouts	2	(4)	Turkey	25	(7)
Other	4	(8)	Other	131	(39)
*Subtotal*^b^	52	(100)	*Subtotal*^b^	340	(100)
*Salmonella*			*Campylobacter*		
Sprouts	11	(18)	Dairy	81	(66)
Nuts-seeds	11	(18)	Chicken	20	(16)
Fruits	6	(10)	Other poultry	5	(4)
Chicken	6	(10)	Molluscs	4	(3)
Eggs	5	(8)	Turkey	4	(3)
Other	23	(37)	Other	8	(7)
*Subtotal*^b^	62	(100)	*Subtotal*^b^	122	(100)
*Listeria*			*E. coli*		
Dairy	13	(62)	Beef	42	(42)
Fruits	3	(14)	Vegetable row crops	30	(30)
Sprouts	3	(14)	Dairy	17	(17)
Vegetable row crops	2	(10)	Fruits	6	(6)
			Game	4	(4)
			Other	2	(2)
*Subtotal*^b^	21	(100)	*Subtotal*^b^	101	(100)
Norovirus			*Clostridium perfringens*		
Molluscs	15	(100)	Beef	28	(37)
			Chicken	17	(23)
			Pork	11	(15)
			Turkey	9	(12)
			Grains-beans	6	(8)
			Other	4	(5)
*Subtotal*^b^	15	(100)	*Subtotal*^b^	75	(100)
*Vibrio*			Norovirus		
Molluscs	8	(100)	Vegetable row crops	17	(23)
			Molluscs	13	(18)
			Other	11	(15)
			Fruits	11	(15)
			Pork	4	(5)
			Other	18	(24)
*Subtotal*^b^	8	(100)	*Subtotal*^b^	74	(100)
***Subtotal***	158	(85)		712	(62)
***Other identified combinations***	28	(15)		439	(38)
***Total identified combinations***	186	(100)		1151	(100)

a This table includes only outbreaks caused by a confirmed single aetiology and a single food category. Outbreaks caused by a sick food handler are excluded.

b The subtotals include all outbreaks caused by a confirmed single aetiology and a single food category for this aetiology, including the non-top food categories.

Of recall-associated outbreaks, approximately 50% (115/226) were multistate outbreaks, compared with only 2% (139/7791) non-recall outbreaks reported (Fig. 1). Forty-five per cent (115/254) of multistate outbreaks were associated with food recalls, with a downward trend from 64% in 2006 to 24% in 2016. For multistate recall-associated outbreaks, 12 215 illnesses, 2556 hospitalisations and 99 deaths were reported. These outbreaks averaged 106 illnesses per outbreak with a 36.8% hospitalisation rate and a 3.7% death rate. In comparison, multistate outbreaks not associated with food recalls had 5112 illnesses, 814 hospitalisations and 9 deaths, with an average of 37 illnesses per outbreak, a 23.1% hospitalisation rate and a 0.9% death rate (Table 1). One hundred and two multistate recall-associated outbreaks were linked to a single food category. Among them, the top food categories recalled were beef (21 in 102 outbreaks, 21%), sprouts (13, 13%), dairy (12, 12%), nuts and seeds (12, 12%) and fruits (11, 11%). In comparison, among 103 multistate outbreaks without recalls that were linked to a single food category, vegetable row crops (24, 23%), seeded vegetables (16, 16%) and fruits (13, 13%) were the most frequent sources.

Among the foods that were the source of at least five multistate recall-associated outbreaks, dairy (12 recalls in 15 outbreaks, 80%; only one recall was linked to a pasteurised product), herbs (4/5, 80%), nuts and seeds (12/15, 80%), beef (21/32, 66%) and sprouts (13/20, 65%) had the largest proportion of outbreaks with recalls. In contrast, seeded vegetables (3/19, 16%), vegetable row crops (7/31, 23%) and chicken (3/9, 33%) had the smallest proportion of multistate outbreaks with recalls. When further limiting this multistate outbreaks sample to the more severe ones (those with at least ten total cases and at least 5% of patients hospitalised), nuts and seeds (6/7, 86%), beef (16/21, 76%) and dairy (5/7, 71%) were the food categories with the biggest proportion of outbreaks with recalls. Chicken (3/6, 50%), vegetable row crops (7/18, 39%) and seeded vegetables (3/17, 18%) were the foods with the smallest proportion among these more severe multistate outbreaks with recalls.

The timing of a recall varied by illness onset and duration of the outbreak. Among 226 recall-associated outbreaks, recalls occurred during the outbreak in 61 (27%), while recalls occurred after the outbreak concluded in 109 (48%) (Table 6). The recall timing also varied by food vehicle. Among the 16 recall-associated sprout outbreaks, seven (44%) had recalls during the outbreak and five (31%) had recalls after the outbreak concluded. In contrast, only three (10%) of mollusc-associated outbreaks had recalls during the outbreak and 20 (67%) had recalls after the outbreak concluded. Dairy and beef followed a pattern similar to that of molluscs. Among dairy products, however, pasteurised cheeses were recalled during the outbreak as often as after the outbreak concluded.

**Table 6.** Timing of recalls by food category, foodborne disease outbreak surveillance system, United States, 2006–2016

Food category	Before outbreak	During outbreak	After outbreak	Inconclusive^a^	Unknown	Total recalls
Aquatic animals						
Fish	1	0	4	1	7	13
Molluscs	2	3	20	0	5	30
Land animals						
Beef	0	10	17	0	5	34
Pork	1	0	2	0	1	5
Chicken	0	4	2	0	0	6
Turkey	0	1	1	0	0	2
Game	0	0	1	0	0	1
Eggs	0	0	3	1	1	5
Dairy	0	7	19	0	7	33
Plants						
Vegetable row crops	0	3	4	0	1	8
Seeded vegetables	2	2	2	0	0	6
Root/underground	0	0	1	0	0	1
Sprouts	0	7	5	1	3	16
Herbs	0	4	0	0	1	5
Grains/beans	0	3	0	0	0	3
Nuts/seeds	0	5	7	0	0	12
Fruits	0	5	8	4	0	17
***Other***^b^	0	0	2	0	3	5
***Single food category***	6	54	98	7	34	199
***Multiple food categories***	0	5	6	0	4	15
***Unknown food category***	0	2	5	0	5	12
***Total recall-associated outbreaks***^c^	6	61	109	7	43	226
***Percentage of recall-associated outbreaks***^d^	3	27	48	3	19	100

a Recalls occurred after the date of first illness onset but it is uncertain if it occurred before the last illness onset.

b The sources of these five recall-associated outbreaks were sindoor (recalled after outbreak), a flavoured drink (recall date unknown), dietary supplements (recall dates unknown for both outbreaks) and seaweed (recalled after outbreak).

c Total number includes all the recall-associated outbreaks caused by a single food category, multiple food categories and unknown food category of any aetiology type (single, multiple, or unknown).

d Proportion of the recall-associated outbreaks by timing in total recall-associated outbreaks.

## Discussion

We identified substantial differences between outbreaks with a food recall and those without in regards to the number of illnesses, proportions of hospitalisations and deaths, leading aetiologies, major food categories, and the proportion that were multistate. The pathogens that caused most recall-associated outbreaks (*Salmonella*, *E*. *coli* and *Listeria monocytogenes*) are routinely identified as pathogens that contaminate commercially distributed foods. Outbreaks caused by the leading sources of non-recall-associated outbreaks, including norovirus, *Campylobacter* and *Clostridium perfringens*, have been frequently linked to food preparation errors or are difficult to identify with current laboratory techniques. A notable exception is molluscs, particularly oysters, for which norovirus contamination typically occurs before harvest due to contaminated water [7].

Outbreaks during the study period due to unpasteurised dairy, beef, molluscs, fruit and sprouts were more likely to have an associated recall than those due to other foods. Some of these foods (unpasteurised dairy, molluscs, sprouts and some fruits) are relatively infrequently consumed by the public, making them easier to identify as the outbreak source [8–10]. Foods like molluscs and some fruits also are relatively easier to trace to their production origin [11, 12]. Foods less commonly associated with recalls included chicken, pork and leafy greens. For some of these foods, the specific source of the outbreak vehicle tends to be difficult to identify. Chicken, for instance, is a food that many Americans eat often, so identifying an epidemiological link to a specific producer of chicken can be challenging [13]. Another reason that may explain why some foods are not frequently recalled is that a recall may not be warranted. Foods with a short shelf life, such as lettuce or spinach, may have been eaten or discarded by the time investigators determine the outbreak source and trace it to its origin. Some technologies, such as whole genome sequencing and blockchain (a type of record-keeping that improves product traceability) could aid investigators in identifying contaminated foods and their sources, which could lead to more recalls of contaminated foods [13–15]. Expediting the epidemiological components of outbreak investigations, particularly case-patient interviews and follow-up, also could improve timeliness [16] and potentially increase the likelihood of a recall.

Multistate outbreaks accounted for half of the recall-associated outbreaks but only 2% of the non-recall outbreaks reported during the study period. Unlike many single state outbreaks, multistate outbreaks are often due to commercially distributed foods and tend to involve a larger number of illnesses, hospitalisations and deaths [2]. Single state outbreaks are less likely to result in a recall for at least two reasons. First, single state outbreaks are often linked to a single restaurant and are caused by improper food handling and preparation practices in the restaurant [17]. Second, single state outbreaks tend to have fewer cases than their multistate counterparts [18], which limits the amount of information local and state investigators have available to identify a food vehicle, making these outbreaks sometimes more difficult to solve.

Among recall-associated outbreaks, approximately one quarter of the recalls occurred during the outbreak, indicating that these recalls might have occurred quickly enough to help prevent additional illnesses. Outbreaks that fell into this category were often due to foods that are either relatively quickly identified, such as sprouts, or have a long shelf life, such as grains and beans, suggesting that recalls might be more easily achievable for some types of foods than others. Recalls are of limited value for preventing disease when the contaminated food has a short shelf life unless the recall occurs very quickly. For foods with a longer shelf life, the public health impact could be greater, presuming the public notification is timely, clear and convincing [19]. Nearly half of the food recalls occurred after the outbreaks had ended; many of these outbreaks were due to molluscs, dairy and fruits, which often have a relatively short shelf life. In these outbreaks, the recall was likely too late to prevent many additional illnesses, but it nevertheless served to notify the public and industry stakeholders that there may have been a food safety issue associated with a particular food company or category. These notifications could inform how attention is focused and resources are directed so that similar outbreaks are less likely to occur.

The findings in this study have at least two limitations. First, FSIS microbiological sampling and subtyping projects are focused on the detection of STEC, *Salmonella*, *Listeria monocytogenes* and *Campylobacter*, so illnesses associated with these pathogens are more likely than illnesses associated with other aetiologies to be linked to FSIS-regulated products (meat, poultry and egg products) and, therefore, recalled. Second, the frequency of recalls occurring after outbreaks have concluded may be an underestimate of situations in which outbreaks were linked with short shelf life food products since FSIS is more likely to recommend a public health alert, rather than a recall, for a potentially harmful product that is no longer commercially available.

The differences between outbreaks associated with a food recall and those that were not help define the types of outbreaks and food vehicles that are likely to lead to a recall. For foods that were less likely to be associated with recalls, such as chicken and leafy greens, an enhanced ability to quickly identify the specific food vehicle in an outbreak investigation could increase the likelihood of a recall. Enhanced food traceability may also speed outbreak investigations and lead to more outbreak-associated recalls. The timing of recalls may affect their impact, which can vary from preventing additional outbreak-associated illnesses to helping develop prevention measures to ensure that similar outbreaks do not occur in the future. A better understanding of how recall timeliness affects outbreak-associated illness numbers could help inform intervention and prevention efforts.

## Data Availability

The FDOSS data are available at National Outbreak Reporting System (NORS) Dashboard | CDC. For more detailed data, one can request it by contacting NORSDashboard@cdc.gov. It is publicly available upon request. And for more info on the type of data collected, one can refer to: Forms & Guidance | National Outbreak Reporting System (NORS) CDC.
